# Prospective appraisal of clinical diagnostic algorithms for hepatocellular carcinoma surveillance in Chinese patients with chronic hepatitis B infection

**DOI:** 10.1038/s41598-024-80257-w

**Published:** 2024-11-22

**Authors:** Henry L. Y. Chan, Yao Hu, Katharina Malinowsky, Kairat Madin, Konstantin Kroeniger, Jinlin Hou, Ashish Sharma

**Affiliations:** 1 The Chinese University of Hong Kong, Hong Kong, Hong Kong Special Administrative Region China; 2 Department of Laboratory Medicine, Huashan Hospital, Shanghai Medical College, Fudan University, Shanghai, China; 3 Microcoat Biotechnologie GmbH, Bernried, Germany; 4 Roche Diagnostics GmbH, Penzberg, Germany; 5 Southern Medical University, Guangzhou, China; 6 Clinical Development and Medical Affairs, Roche Diagnostics International AG, Rotkreuz, Switzerland

**Keywords:** Hepatocellular carcinoma, Hepatitis B virus, Diagnostic algorithms, GAAD, GALAD, Hepatocellular carcinoma, Diagnostic markers, Cancer screening

## Abstract

Hepatocellular carcinoma (HCC) is often detected at advanced stages among patients with hepatitis B virus (HBV), underscoring the urgency for more precise surveillance tests. Here, we compare the clinical performance of the novel - GAAD (gender [biological sex], age, alpha-fetoprotein [AFP], protein-induced by vitamin K absence-II [PIVKA-II]) and GALAD (gender [biological sex], age, AFP, *Lens-culinaris* AFP [AFP-L3]), PIVKA-II) algorithms to assess the utility of AFP-L3 for distinguishing HCC from benign chronic liver disease (CLD) in Chinese patients with predominantly chronic HBV infection. Eligible adults were enrolled, and biomarkers were measured using Elecsys (Cobas) or µTASWAKO assays. In total, 411 participants provided serum samples (HCC, *n* = 176 [early-stage, *n* = 110]; CLD, *n* = 136; specificity *n* = 101). HBV was the underlying disease etiology for most participants (HCC, 95%; benign CLD, 72%). For GAAD (Cobas), GALAD (Cobas), and GALAD (µTASWAKO), AUCs were 93.1% (95% CI: 90.0–96.2), 93.2% (90.0–96.3), and 92.7% (88.4–96.9) for early-stage, and 95.6% (93.6–97.6), 95.6% (93.6–97.7), and 95.8% (93.2–98.3) for all-stage HCC, versus CLD, respectively. Interestingly, both GAAD and GALAD algorithms demonstrated comparable diagnostic performance regardless of disease etiology (HBV vs. non-HBV), presence of cirrhosis, geographic region, and within pan-tumor specificity panels (*p* < 0.001), indicating AFP-L3 may have a negligible role in HCC surveillance.

**Supplementary Information:**

The online version contains supplementary material available at 10.1038/s41598-024-80257-w.

## Introduction

Liver cancer remains a global health concern, with incidence projected to rise approximately 55% by 2040, in line with the growing population^1^. In particular, high incidence and mortality rates are observed in Asia, with approximately 73% of all new liver cancer cases and associated deaths reported in Asia alone;^1^ the majority of these (60%) are reported in China^2^. Similarly, Hong Kong has a high prevalence of liver cancer, with hepatocellular carcinoma (HCC) the third most common cause of cancer-associated deaths^3^. Risk factors for HCC include alcohol-related cirrhosis and metabolic dysfunction-associated steatohepatitis (MASH)^4–6^. However, viral hepatitis infection, primarily hepatitis B virus (HBV), represents a major risk factor for chronic liver disease (CLD) and subsequent development of HCC in around half of all HCC cases^7,8^.

Despite nationwide vaccination programs and therapeutic developments, China has a high intermediate prevalence of HBV (6.89%), accounting for one third of all HBV infections and deaths globally^9,10^. Notably, there is a high prevalence of HBV in women of child-bearing age in China, resulting in high levels of perinatal transmission^10^. As a result, ~ 60% of HCC cases reported in China have HBV-related etiology, which confers a more malignant phenotype of HCC, characterized by rapid progression^7^. HBV contributes to the development of HCC through multiple mechanisms, including chronic inflammation and chromosomal instability through integration of HBV DNA (e.g., insertional mutagenesis in proto-oncogenes and tumor suppressors, and expression of mutant HBV proteins)^11,12^. These epigenetic changes are induced by the HBV-encoded oncogene X protein (HBx), which contributes to carcinogenesis by modifying the transcription of DNA methyltransferase and inducing cellular oxidative stress^12–14^.

Furthermore, the increasing incidence of obesity and obesity-related diseases, such as type 2 diabetes, in Asian populations has resulted in an increased prevalence of metabolic dysfunction-associated fatty liver disease (MAFLD), which is associated with an increased risk of HCC^4^. In addition, MASH, defined by lobular inflammation and hepatocyte ballooning, was identified in 63% of patients with metabolic dysfunction-associated steatotic liver disease (MASLD) in an Asian multi-center cohort and was associated with an increased risk of fibrosis progression^4^. As the coexistence of both chronic HBV infection and MAFLD is becoming increasingly common in the Chinese population, the occurrence of HCC in these patients is of significant concern^17^.

The high prevalence of HBV and the associated risk factors for HCC facilitated the implementation of surveillance programs for at-risk individuals, including those with viral hepatitis and/or cirrhosis, with the intention of detecting HCC in earlier stages. Timely diagnosis of HCC is essential to improve prognosis as curative therapies are more feasible for early-stage disease^18^), and it has been shown to account for the increased five-year survival rate observed for early- (> 70%) compared with advanced-stage HCC (< 20%)^19–21^.

In China, surveillance using ultrasound, with or without profiling of the biomarker, α-fetoprotein (AFP), is recommended every 6 months for high-risk individuals^22–24^. However, ultrasound, AFP and other serum biomarkers, such as protein induced by vitamin K absence or antagonist-II (PIVKA-II) (previously des-*γ* carboxyprothrombin [DCP]) and *Lens culinaris* agglutinin-reactive fraction of AFP (AFP-L3), have demonstrated limited sensitivity for detection of HCC when used as standalone indicators^22–25^.

The development of diagnostic algorithms that combine patient demographic characteristics with the measurement of multiple serum biomarkers have further improved early detection of HCC^26–29^. One such algorithm, GALAD (gender [biological sex], age, α-fetoprotein [AFP], *Lens culinaris* agglutinin-reactive fraction of AFP [AFP-L3], protein induced by vitamin K absence or antagonist-II [PIVKA-II]), combines gender (sex at birth) and age, with the measurement of three serum biomarkers (AFP, AFP-L3, and PIVKA-II), and has demonstrated superior clinical performance versus single biomarkers for the differentiation of HCC and CLD^29–31^. This is reflected in the current recommendations in China, where serum AFP combined with AFP-L3 and PIVKA-II are recommended to improve the detection of early-stage liver cancer^32^.

Notably, in populations with a high burden of viral hepatitis, such as Chinese and other Asian populations, the impact of antiviral therapies on serum biomarkers and their diagnostic value must be considered. For example, AFP has been shown to have high sensitivity for the detection of HCC, with optimal cut-off values lower in those receiving antiviral therapy for chronic viral hepatitis compared with those not receiving antiviral therapy^33,34^. Furthermore, AFP has also demonstrated improved clinical performance in patients with hepatitis C virus (HCV) and preserved liver function as a result of antiviral therapy^35^, suggesting that AFP-L3 may no longer meaningfully contribute to the detection of HCC, either as a standalone biomarker or as part of the GALAD diagnostic algorithm. As such, the GAAD (gender [biological sex], age, α-fetoprotein [AFP], protein induced by vitamin K absence or antagonist-II [PIVKA-II]) algorithm was developed to assess the utility of AFP-L3 for distinguishing early-stage HCC from benign chronic liver disease^36^. Recently, the National Health Commission of the People’s Republic of China noted that the simplified GAAD diagnostic algorithm has a similar diagnostic performance to GALAD (highest level evidence, strong recommendation)^37^. However, there is currently no comparative study evaluating the efficacy of these diagnostic algorithms within a Chinese population. Therefore, in this study, we compared the clinical performance of GAAD and GALAD algorithms for the detection of HCC versus benign CLD in a Chinese subset population with patients with predominantly chronic hepatitis from an international, multicenter, case–control study.

## Methods

### Study population

This study was conducted within a case–control cohort of individuals aged ≥ 18 years, who were prospectively enrolled at two centers in the People’s Republic of China and Hong Kong Special Administrative Region of China, as part of the STOP-HCC-MCE study^38^. To be eligible for inclusion for clinical performance analysis, participants had a first-time HCC diagnosis, confirmed by either radiology according to international guidelines (or a ≥ 1 cm lesion showing arterial-phase hyper enhancement in combination with washout appearance and/or capsule by quadruple-phase computed tomography scan or multiphase contrast-enhanced magnetic resonance imaging^22^) or by positive pathology within 6 months of enrolment. Briefly, the BCLC staging system was used to categorize patients with HCC into one of the following disease stages: very early HCC (stage 0; single nodule ≤ 2 cm without vascular invasion or extrahepatic spread in a asymptomatic patient with preserved liver function); early HCC (stage A; single nodule or ≥ 3 nodules < 3 cm without macrovascular invasion or extrahepatic spread in an asymptomatic patient [performance status (PS) 0]); intermediate HCC (stage B; multifocal HCC with no vascular invasion or extrahepatic spread in an asymptomatic patient with preserved liver function [PS 0]); advanced HCC (stage C; patients with vascular invasion or extrahepatic spread presenting with PS ≥ 2 and preserved liver function); end-stage (stage D; major cancer-related symptoms [PS > 2] and/or impaired liver function without the option of liver transplant due to HCC burden or non–HCC–related factors).

Eligible CLD controls comprised of at-risk patients with either cirrhotic or non-cirrhotic CLD (resulting from HBC or HCV infection, alcoholic liver disease, or MASH) and confirmed absence of HCC by imaging within the previous 12 months. Exclusion criteria for both the HCC and CLD groups included any other form of cancer (excluding non-melanoma skin cancer), recurrent HCC, or previous or current treatment for HCC, glomerular filtration rate < 60 mL/min/1.73 m^2^, or treatment with anti-vitamin K coagulant therapy (e.g., warfarin, phenprocoumon, or acenocoumarol).

For the specificity panel, samples from patients with other malignancies (e.g., colorectal cancer) and other benign diseases (e.g., liver cysts, ulcerative colitis, Crohn’s disease, and rheumatoid arthritis) were included. Patients with glomerular filtration rate < 60 mL/min/1.73 m^2^, cholangiocarcinoma, and/or pancreatic cancer were excluded from this analysis due to low sample numbers.

All approvals were obtained from the relevant ethics committees and institutional review boards for all study sites involved in sample collection. All methods were performed in accordance with relevant local guidelines and regulations. Each participant provided informed consent before enrolment, and local rules regarding informed consent for the subsequent use of collected samples were followed.

### Serum sample collection and assessment

Serum samples were collected ≥ 1 day before any planned procedures requiring general anesthesia and were stored at − 70 °C at the collection sites. Serum levels of PIVKA-II, AFP, and AFP-L3 were measured using Elecsys assays on the Cobas e 601 analyzer, or µTASWAKO assays on the Fujifilm Micro Total Analysis System WAKO analyzer. Predefined cut-off values used for the detection of HCC versus benign CLD were as follows: 20 ng/mL for AFP (Elecsys); 2.3 ng/mL for AFP-L3 (Elecsys); 28.4 ng/mL for PIVKA-II (Elecsys); 2.57 (range 0–10) for GAAD (Cobas); 2.47 (range 0–10) for GALAD (Cobas), and − 0.63 for GALAD (µTASWAKO). Additional cut-off values for GALAD (µTASWAKO) were also assessed, corresponding to GAAD (Cobas) specificity of 90%.

### GAAD and GALAD (Cobas) algorithm development

The GAAD and GALAD (Cobas) algorithm development process has been previously described^38^. In summary, multivariate analyses were carried out to identify the best-performing panel of biomarkers for HCC detection using two methods, lasso regression (no fixed panel size) and exhaustive search with logistic regression (fixed panel size of two to four biomarkers). The full data set from the previous algorithm development study (STOP-HCC-ARP) was used to train logistic regression models for the two top-performing clinical algorithms (GAAD and GALAD), with diagnosis of HCC (Barcelona Clinic Liver Cancer [BCLC]-stage independent) as the predictor variable^38^. The cut-offs were calculated on the predictor variable from 90% specificity.

### Statistical analysis

The clinical performance of the GAAD and GALAD algorithms, and that of the individual biomarkers alone, were compared using receiver operating characteristic (ROC) analysis and area under the curve (AUC) values. For sensitivity and specificity analyses, the derived 95% confidence intervals (CIs) were calculated from the binomial distribution using the Clopper–Pearson method. P-values comparing non-inferiority of AUCs were calculated using the H0 hypothesis AUC (Test1) < AUC (Test 2)-0.01 with a studentized bootstrap approach using *N* = 1000 bootstrap replicates. For the specificity panel, p-values were calculated using binomial tests with Bonferroni correction for every marker using the cut-offs listed previously, and a specificity value ≥ 90%^38^; disease groups with fewer than 5 patient samples were excluded from analysis.

## Results

### Study population

**Fig. 1:**
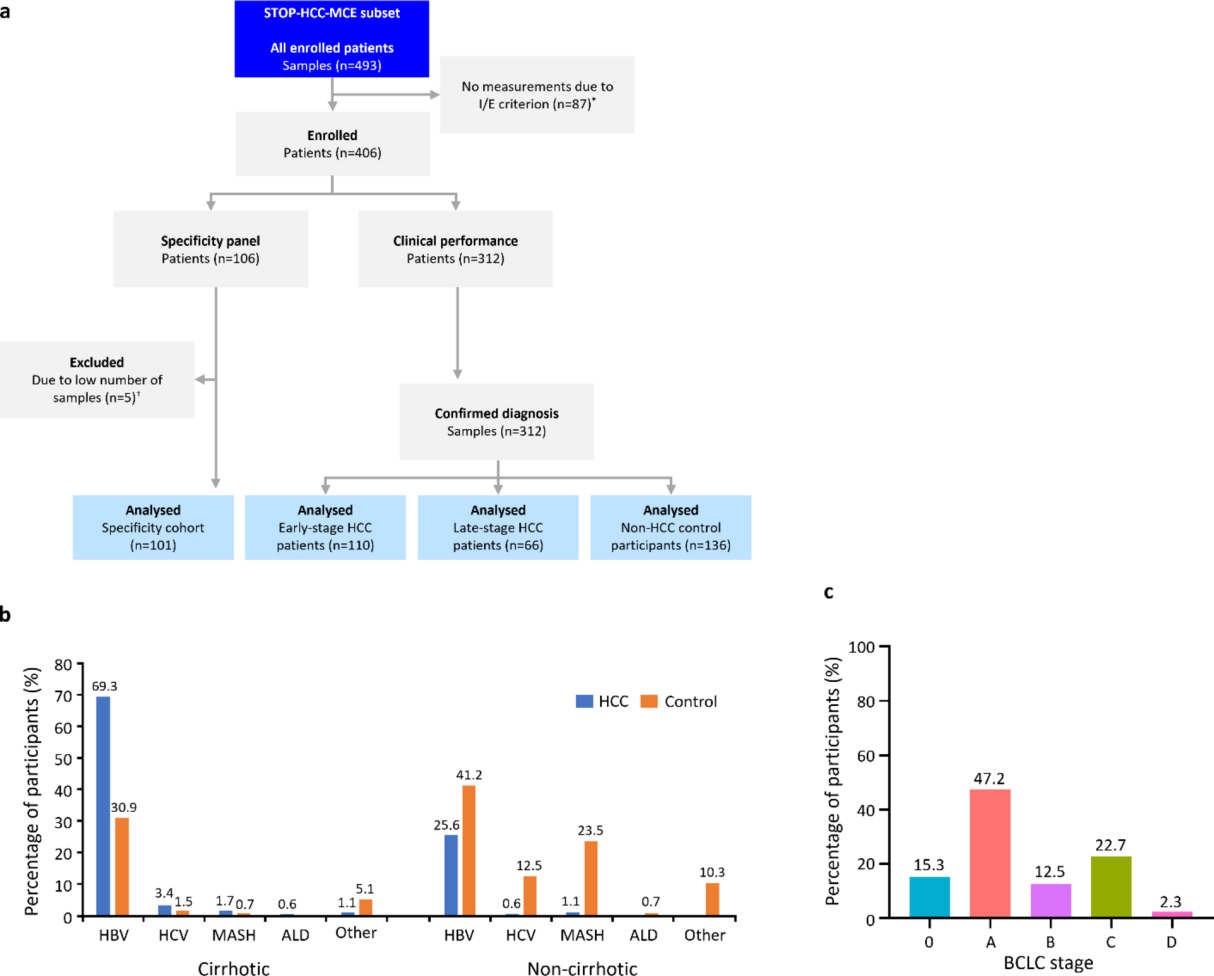
Study design for the Chinese cohort (Subset of STOP-HCC-MCE) (**A**) and baseline disease characteristics (**B**,**C**). *Reasons for exclusion were: missing required laboratory parameter data (*n* = 37); GFR < 60 mL/min/1.73 m^2^ (*n* = 19); other cancer diagnosis (*n* = 11); non-confirmed diagnosis (*n* = 8); incomplete/incorrect sample processing (*n* = 4); current/previous treatment with anti-vitamin K coagulant therapy (*n* = 3); written informed consent not obtained (*n* = 2); sponsor decision (*n* = 2) and recurrent HCC (*n* = 1). ^†^Participants were excluded due to low number of samples (*n* < 5).

In total, 312 Chinese participants enrolled in this case–control study provided confirmed diagnostic samples (HCC, *n* = 176; benign CLD, *n* = 136) for the clinical performance analysis, and an additional 101 participants were included in the specificity panel cohort (Fig. 1).

In the clinical performance cohort, the majority of patients were male (total HCC, 85.8%; benign CLD controls, 69.1%), and the mean age in the HCC group and the benign CLD group, respectively, was 55.4 years and 46.5 years (Table 1). HBV was the underlying disease etiology for the majority of participants (HCC, 94.9%; benign CLD, 72.1%) (Fig. 1b), and underlying cirrhosis was present in 73.3% and 33.8% of the HCC group and the benign CLD group, respectively. BCLC stage varied across the HCC group, with most participants classified as having early-stage HCC (BCLC 0–A, 62.5%) compared with late-stage HCC (BCLC B–D, 37.5%) (Fig. 1c). The proportion of participants receiving antiviral therapy was similar between groups (HCC, 63.1%; benign CLD, 61.0%) (Table 1). Baseline demographics and characteristics according to clinical study site are shown in Supplementary Table S1.

**Table 1:** Demographic, disease, and tumor characteristics of the Chinese subpopulation cohort.

Participant characteristics	Early-stage HCC (*n* = 110)	Late-stage HCC (*n* = 66)	Total HCC (*n* = 176)	Benign CLD controls (*n* = 136)
Age, years; mean (SD)	56 (11.7)	54.6 (9.1)	55.4 (10.8)	46.5 (11.3)
Sex, n (%)				
Male	96 (87.3)	55 (83.3)	151 (85.8)	94 (69.1)
Female	14 (12.7)	11 (16.7)	25 (14.2)	42 (30.9)
Ongoing antiviral therapy, n (%)	78 (70.9)	33 (50.0)	111 (63.1)	83 (61.0)
Liver biochemistry (U/L), median (IQR)				
AST	30.5 (23.0–40.8)	59.5 (37.5–128.0)	37.0 (25.8–57.0)	23.0 (20.0–33.0)
ALT	29.0 (23.0–40.0)	46.0 (30.5–68.8)	34.0 (24.0–50.0)	25.0 (18.0–38.3)
PT-INR, n (%)*				
1	110 (100)	65 (98.5)	175 (99.4)	0
2	0	0	0	0
3	0	1 (1.5)	1 (0.6)	0
Ascites, n (%)				
Mild	7 (6.4)	18 (27.3)	25 (14.2)	0
Moderate to severe	1 (0.9)	10 (15.2)	11 (6.3)	0
None	102 (92.7)	38 (57.6)	140 (79.5)	136 (100)
Serum albumin (g/L), median (IQR)	39.0 (35.8–41.0)	35.3 (30.6–39.0)	38.0 (33.5–40.5)	44.0 (40.0–46.5)
Serum total bilirubin (µmol/L), median (IQR)	13.5(10.8–19.0)	17.1 (12.0–29.9)	14.6 (11.1–21.0)	12.0 (9.0–16.2)
MELD score, median (IQR)	7 (6.0–8.8)	8 (7.0–10.0)	7 (7–9)	7 (6–7)
ALBI score, median (IQR)	-2.6 (-2.8–2.3)	-2.3 (-2.6–1.7)	-2.43 (-2.69–2.04)	-3.03 (-3.27–2.69)
ALBI grade, n (%)				
1	50 (45.5)	14 (21.2)	64 (36.4)	109 (80.1)
2	59 (53.6)	44 (66.7)	103 (58.5)	27 (19.9)
3	1 (0.9)	8 (12.1)	9 (5.11)	0
Tumor characteristics*				
PST, n (%)				NA
0	106 (96.4)	34 (51.5)	140 (79.5)	NA
1	4 (3.6)	27 (40.9)	31 (17.6)	NA
2	0	5 (7.6)	5 (2.8)	NA
Child–Pugh class, n (%)				NA
A	101 (91.8)	41 (62.1)	142 (80.7)	NA
B	9 (8.2)	21 (31.8)	30 (17.0)	NA
C	0	4 (6.1)	4 (2.3)	NA
Nodule number, n (%)				NA
1	105 (95.5)	6 (9.1)	111 (63.1)	NA
2	4 (3.6)	11 (16.7)	15 (8.5)	NA
3	1 (0.9)	5 (7.6)	6 (3.4)	NA
≥3	0	44 (66.7)	44 (25.0)	NA
HCC characteristics, n (%)				NA
Large multinodular	0	28 (42.4)	28 (15.9)	NA
Portal vein invasion or EHS (N1, M1)	0	36 (54.5)	36 (20.5)	NA
Single < 2 cm	31 (28.2)	0	31 (17.6)	NA
Single or ≤ 3 nodules ≤ 3 cm	79 (71.8)	2 (3.0)	81 (46.0)	NA
Size of index lesion (cm); mean (IQR)	3.3 (1.8–4.3)	9.0 (6.1–11.7)	5.4 (2.2–8.5)	NA
Minimum–maximum	0.9–11.3	0.7–18.4	0.7–18.4	NA

*Tumor characteristics are not applicable to all participants in the benign CLD group.

*ALBI* albumin-bilirubin,* ALT* alanine aminotransferase,* AST* aspartate aminotransferase,* CLD* chronic liver disease,* EHS* extrahepatic spread,* HCC* hepatocellular carcinoma,* IQR* interquartile range,* MELD* Model for End-Stage Liver Disease,* NA* not applicable,* PST* performance status,* PT-INR* prothrombin international normalized ratio,* SD* standard deviation.

### Method comparison and specificity panel analysis

**Fig. 2:**
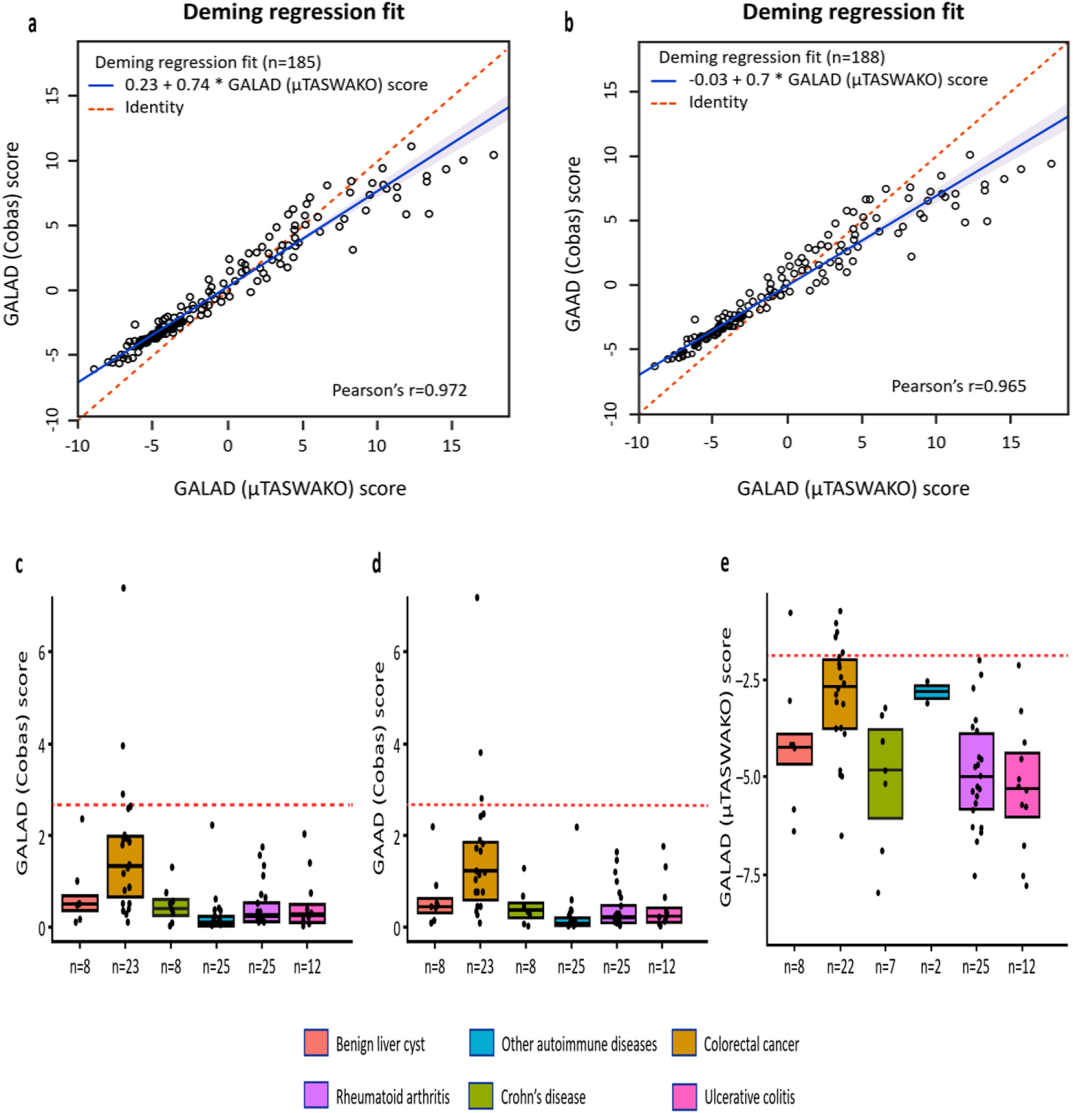
Method comparison and specificity panel—weighted Deming regression fit of agreement between GALAD (Cobas) and GALAD (µTASWAKO) (**A**) and GAAD (Cobas) and GALAD (µTASWAKO) (**B**). Specificity panel data across disease groups for GAAD (Cobas) (**C**), GALAD (Cobas) (**D**), and GALAD (µTASWAKO) (**E**). The percentage of participants for each etiology does not sum up to 100% as some patients had multiple etiologies at baseline.

Based on weighted Deming regression analyses, excellent analytical method agreement was observed between GALAD (µTASWAKO) and GAAD (Cobas) (Pearson’s *r* = 0.965; *p* < 0.001) and GALAD (Cobas) (Pearson’s *r* = 0.972; *p* < 0.001) algorithms in this Chinese cohort (Fig. 2a, b).

The specificity panel analysis is depicted in Fig. 2c–e. Low profiles of GAAD (Cobas), GALAD (Cobas), and GALAD (µTASWAKO) scores were observed across the disease groups available for analysis (i.e., benign liver cysts, colorectal cancer, Crohn’s disease, other autoimmune diseases, rheumatoid arthritis, and ulcerative colitis), with all upper interquartile range values below the established HCC cut-offs, indicating high algorithmic panel specificity for HCC diagnosis.

### Clinical performance

**Fig. 3:**
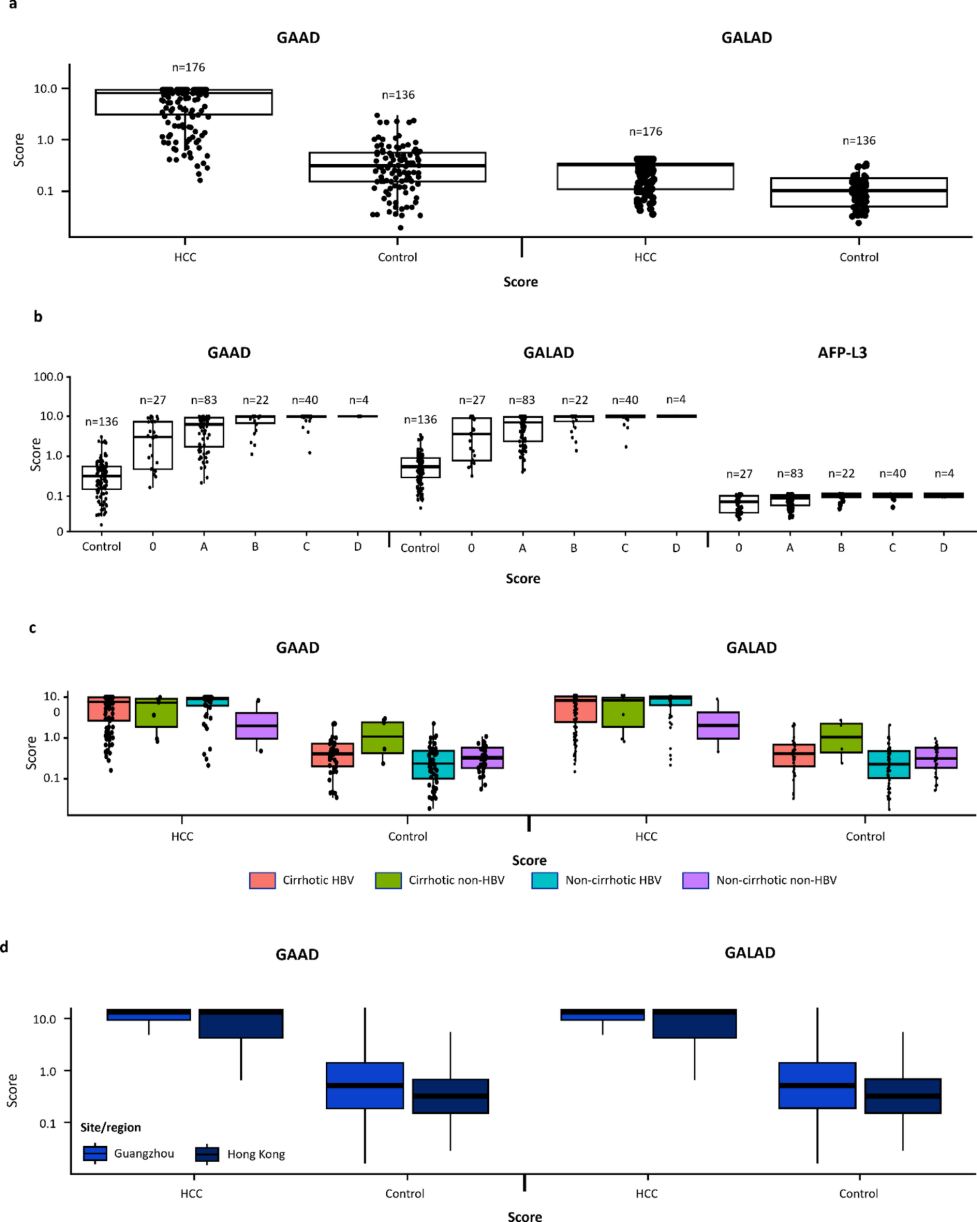
Distribution of GAAD and GALAD (Cobas) scores in HCC cases and benign CLD controls (**A**), and according to BCLC stage (**B**), etiology (**C**) and clinical study site (**D**).

Both GAAD (Cobas) and GALAD (Cobas) algorithm scores effectively differentiated between HCC and benign CLD controls, irrespective of HCC disease stage, viral or non-viral etiology, or study center (Fig. 3a–d). The distribution of GAAD and GALAD algorithm scores was similar for both HCC and benign CLD controls, and according to HCC disease stage, etiology, and study center.

**Fig. 4:**
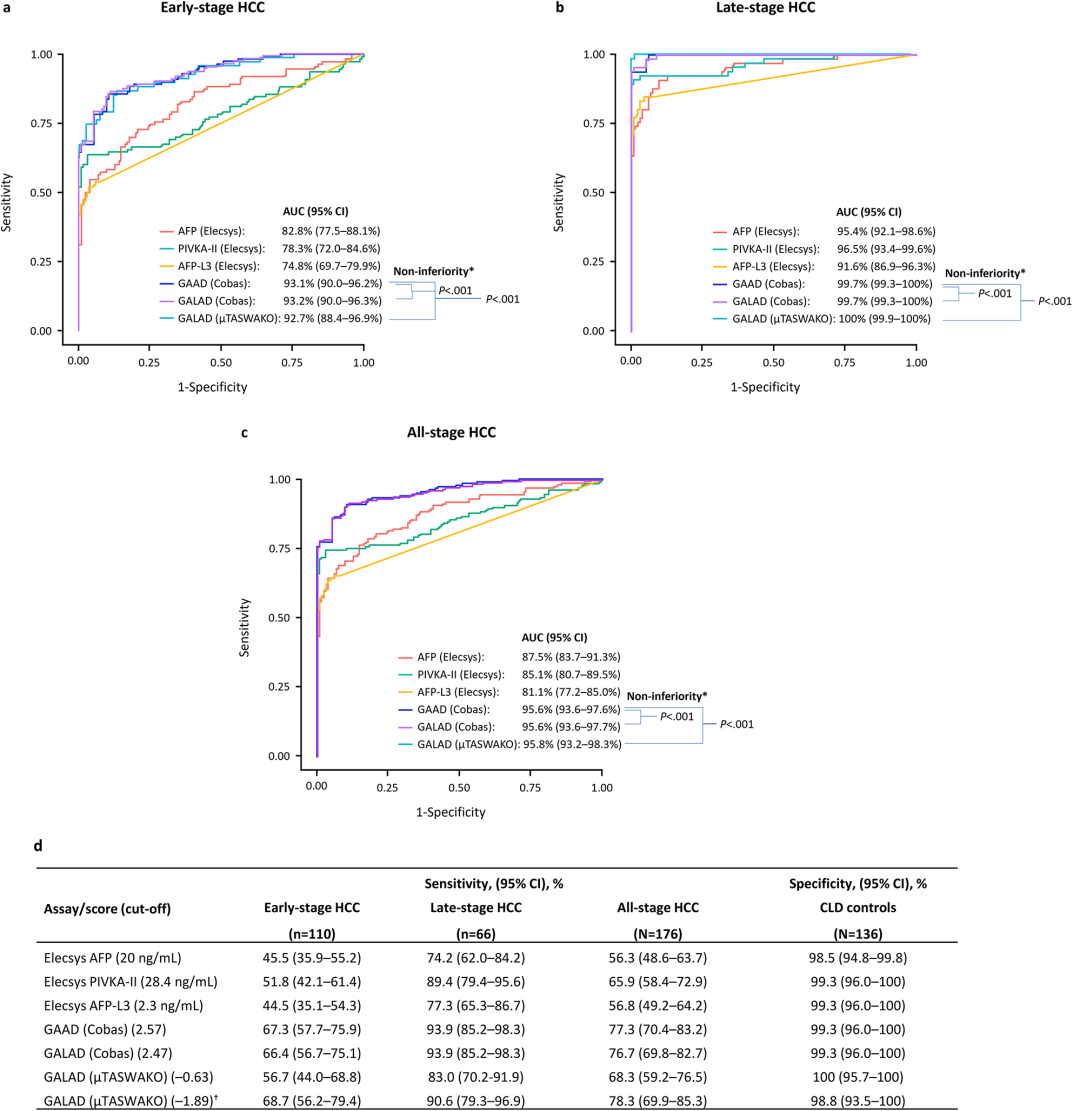
Clinical performance of GAAD (Cobas) versus GALAD (Cobas and µTASWAKO), vs. three serum biomarkers (AFP, PIVKA-II, and AFP-L3) in HCC in early-stage (**A**), late-stage (**B**) and all-stage (**C**) HCC. Sensitivities and specificities are shown in table (**D**). *AUC is not significantly worse by 1%. ^†^Cut-off values correspond to the matching GAAD (Cobas) specificity of 90%.* AFP* alpha fetoprotein,* AFP-L3* Lens culinaris agglutinin-reactive fraction of AFP,* GAAD* Gender (biological sex), Age, AFP, PIVKA-II,* GALAD* Gender (biological sex), Age, AFP-L3, AFP, PIVKA-II,* HCC* hepatocellular carcinoma,* PIVKA-II* protein induced by vitamin K absence-II,* PPV* positive predictive value.

The GAAD (Cobas) and GALAD (Cobas) algorithms demonstrated similar performance in terms of AUC for differentiating between HCC (early-, late- and all-stage) and benign CLD (Fig. 4). For GAAD (Cobas), GALAD (Cobas), and GALAD (µTASWAKO) respectively, AUCs were 93.1% (95% CI: 90.0–96.2), 93.2% (95% CI: 90.0–96.3), and 92.7% (95% CI: 88.4–96.9) for early-stage HCC, 99.7% (95% CI: 99.3–100), 99.7% (95% CI: 99.3–100), and 100% (95% CI: 99.9–100) for late-stage HCC, and 95.6% (95% CI: 93.6–97.6), 95.6% (95% CI: 93.6–97.7), and 95.8% (95% CI: 93.2–98.3) for all-stage HCC, versus benign CLD (Fig. 4a–c). Sensitivity for GAAD (Cobas) and GALAD (Cobas), respectively, was 67.3% and 66.4% for early-stage HCC, 93.9% for both algorithms for late-stage HCC, and 77.3% and 76.7% for all-stage HCC at a specificity of 99.3% for benign CLD (Fig. 4d). For GALAD (µTASWAKO), two cut-offs, − 0.63 and − 1.89 (to mimic 90% specificity with GAAD [Cobas]), were used with respective sensitivities of 56.7% and 68.7% for early-stage HCC, and 68.3% and 78.3% for all-stage HCC, at specificities of 100% and 98.8% for benign CLD, respectively (Fig. 4d). GAAD (Cobas) was non-inferior (within a 1% margin) compared with GALAD (Cobas) and GALAD (µTASWAKO) across HCC stages (*p* < 0.001).

**Fig. 5:**
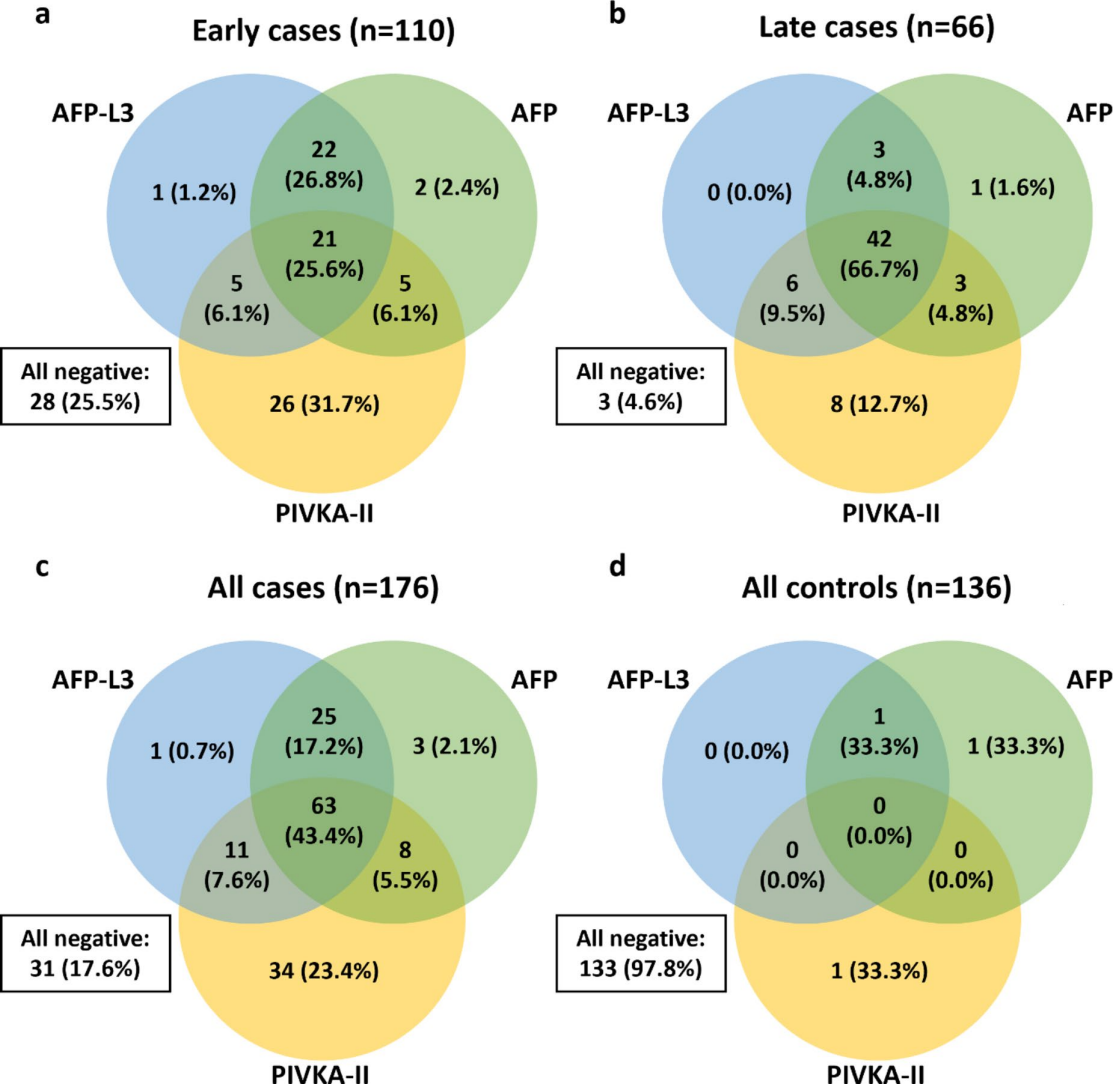
Venn diagram to illustrate the number of early-stage, late-stage, and all-stage cases, or controls detected by the single markers (Elecsys AFP, Elecsys AFP-L3, Elecsys PIVKA-II).

The detection of different HCC stages and benign CLD controls using individual biomarkers is shown in Fig. 5. The clinical performance of the GAAD (Cobas), GALAD (Cobas), and GALAD (µTASWAKO) algorithms was generally superior versus the individual serum biomarkers (AFP, AFP-L3, and PIVKA-II) alone. When each individual marker was used independently for surveillance, AFP-L3 successfully detected an additional early-stage HCC case that was missed by both AFP and PIVKA-II. Conversely, when AFP and PIVKA-II were combined in an algorithm, GAAD detected six additional early-stage and three additional late-stage HCC cases that were missed by GALAD (µTASWAKO), while displaying the same specificity for the CLD controls (Table 2).

**Table 2:** Contingency table for GAAD (Cobas) compared with GALAD (Cobas) and GALAD (µTASWAKO).

Stage		GAAD (Cobas)+	GAAD (Cobas)−	Missing	Total
Early-stage	GALAD (Cobas)+	39	2	1	42
	GALAD (Cobas)−	0	22	0	22
	Missing	0	0	3	3
	Total	39	24	4	67
Late-stage	GALAD (Cobas)+	40	0	2	42
	GALAD (Cobas)−	0	2	0	2
	Missing	0	0	9	9
	Total	40	2	11	53
All-stage	GALAD (Cobas)+	79	2	3	84
	GALAD (Cobas)−	0	24	0	24
	Missing	0	0	12	12
	Total	79	26	15	120
Early-stage	GALAD (µTASWAKO)+	36	0	2	38
	GALAD (µTASWAKO)−	**6**	22	1	29
	Missing	0	0	0	0
	Total	42	22	3	67
Late-stage	GALAD (µTASWAKO)+	36	0	8	44
	GALAD (µTASWAKO)−	**3**	2	0	5
	Missing	3	0	1	4
	Total	42	2	9	53
All-stage	GALAD (µTASWAKO)+	72	0	10	82
	GALAD (µTASWAKO)−	**9**	24	1	34
	Missing	3	0	1	4
	Total	84	24	12	120

GAAD+ (Cobas) corresponds to patients with a GAAD (Cobas) score of ≥ 2.57; GAAD− (Cobas) corresponds to patients with a GAAD (Cobas) score of < 2.57; GALAD+ (Cobas) corresponds to patients with a GALAD (Cobas) score of ≥ 2.47, and GALAD- (Cobas) corresponds to patients with a GALAD (Cobas) score of < 2.47.

GAAD, gender (biological sex), age, AFP, AFP-L3, PIVKA-II; GALAD, gender (biological sex), age, AFP, AFP-L3, PIVKA-II.

Cases that were detected by GAAD (cobas) but were not detect by GALAD algorithms are in bold.

**Fig. 6:**
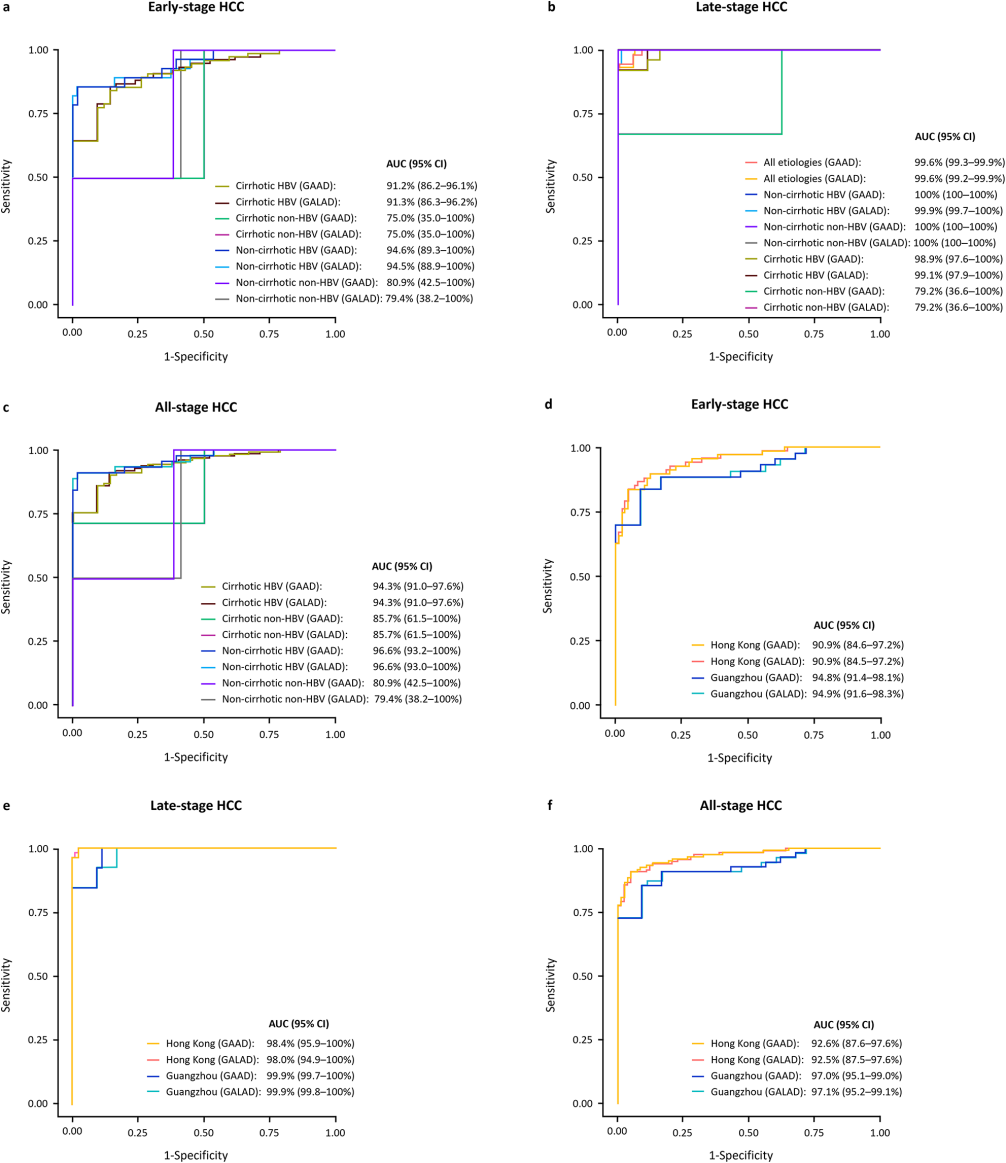
Clinical performance of GAAD and GALAD (Cobas) algorithms for the detection of early-stage (**A**), late-stage (**B**) and all-stage HCC (**C**) according to underlying etiology and early stage (**D**), late-stage (**E**) and all-stage (**F**) according to clinical study site.

The clinical performance of the different algorithms and individual biomarkers at pre-defined cut-offs is shown in Supplementary Table S2. The clinical performance of the different algorithms and individual biomarkers at specified sensitivities and specificities is shown in Supplementary Tables S3 and S4, respectively. The clinical performance of the GAAD (Cobas) and GALAD (Cobas) algorithms was similar for both HBV and non-HBV etiologies, with or without presence of cirrhosis (Fig. 6a–c). Numerically higher AUCs for both algorithms were observed in the HBV versus non-HBV etiology subset and in the non-cirrhotic vs. cirrhotic subset. For the detection of early-stage HCC, AUCs for GAAD and GALAD were 91.2–94.6% in participants with HBV etiology, and 75.0–80.9% in participants with non-HBV etiologies (Fig. 6a). For the detection of late-stage HCC, AUCs for both GAAD and GALAD were 98.9–100% in participants with HBV, and 79.2–100% in participants with non-HBV etiologies (Fig. 6b). For the detection of all-stage HCC, AUCs for both GAAD and GALAD were 94.3–96.6% in participants with HBV and 79.4–85.7% in participants with non-HBV etiologies (Fig. 6c). The clinical performance of the GAAD (Cobas) and GALAD (Cobas) algorithms was also similar between clinical testing sites in Guangzhou and the Hong Kong Special Administrative Region of China in early-, late-, and all-stage HCC participants (Fig. 6d–f).

## Discussion

The National Health Commission of the People’s Republic of China recently updated the guidelines for the diagnosis and treatment of primary liver cancer, stating that the simplified GAAD algorithm demonstrated a similar diagnostic performance to the GALAD algorithm^37^. However, this has previously not been demonstrated in a Chinese population. To the best of our knowledge, this is the first study to show that the clinical performance of the GAAD (Cobas), GALAD (Cobas), and GALAD (µTASWAKO) algorithms was comparable for the differentiation of HCC from benign CLD in a Chinese subpopulation. The improved clinical performance was also observed regardless of HCC disease stage, disease etiology, or clinical site. Additionally, GAAD (Cobas), GALAD, (Cobas), and GALAD (µTASWAKO) algorithms showed greater sensitivity for HCC across all disease stages when compared with individual serum biomarkers (AFP, AFP-L3, and PIVKA-II) alone. The specificity panel demonstrated that the GAAD (Cobas), GALAD (Cobas), and GALAD (µTASWAKO) algorithms were specific for HCC diagnosis, and that markers were not upregulated in other cancers or benign diseases, therefore indicating an accurate HCC diagnosis. Notably, GAAD detected six additional early-stage and three additional late-stage HCC cases that were missed by GALAD (µTASWAKO). GAAD and GALAD also reported equally high specificities; this is of great clinical relevance indicating that GAAD can serve as an effective surveillance strategy compared with GALAD, while maintaining a low false positive rate that is similar to GALAD.

Since China accounts for almost two thirds of all HCC cases reported in Asia, with an incidence rate of 18.2 per 100,000 population, and a mortality rate of 17.2 per 100,000, early HCC detection in the Chinese population is vital^2^. As such, the Working Group on Obesity in China recommended lower BMI cut-offs for obesity compared with the World Health Organization. The high prevalence of HBV in China, often acquired through perinatal transmission, significantly contributes to the high incidence of HCC, with more than half of all HCC cases in China reported to have HBV-related etiology^7,8^. Additionally, the increasing incidence of obesity and obesity-related diseases in China, including MASLD and MASH, further contributes to the high level of HCC observed in this population^2,15,16^. As the feasibility of curative therapies for HCC is highly dependent on the disease stage at the time of diagnosis, preventative interventions, such as vaccination against HBV and HCC surveillance programs, are crucial^22,24,39^. Multiple clinical studies have demonstrated the benefit of HCC surveillance among those with chronic HBV in China. One such study that assessed the effectiveness of liver cancer screening in participants with chronic HBV (*n* = 5,581) in China, found that a higher proportion of those undertaking 6-monthly surveillance with AFP and ultrasound were diagnosed with early-stage liver cancer (29.6%) compared with those undergoing no surveillance (6.0%)^40^. Similarly, another study of participants with HBV or a history of chronic hepatitis (*n* = 18,816) in Shanghai found that, despite a low adherence of 58.2%, biannual screening with AFP and ultrasound reduced HCC mortality by 37%^41^. Furthermore, biannual screening with AFP enabled detection of most HCC tumors at a resectable stage and significantly improved survival rates at 1, 3, and 5 years, compared with no surveillance^41^. Therefore, it is imperative that patients are diagnosed as early as possible to maximize survival outcomes^7^.

For the detection of early-stage HCC, the GAAD (Cobas) and GALAD (Cobas) algorithms achieved sensitivities of 67.3% and 66.4% (each at specificities of 99.3%), respectively, and the GALAD (µTASWAKO) algorithm displayed a sensitivity of 68.7% (specificity 98.8%) at a cut-off of − 1.89. These data suggest that, as the performance of the GAAD algorithm (without the AFP-L3 serum biomarker) was comparable with the GALAD algorithm (Cobas or µTASWAKO), the incorporation of AFP-L3 into diagnostic algorithms may not be essential for the detection of early-stage HCC.

A reduced number of serum biomarkers needed for a sensitive and specific diagnostic algorithm, as seen with GAAD (Cobas), may be beneficial to healthcare systems. It would require fewer samples and assay workups to achieve the same outcomes, thereby reducing economic burden and assay lead-times^42^. Multiple studies have also compared the cost-effectiveness of GAAD with other HCC surveillance modalities. One such study found that both GAAD and GALAD combined with ultrasound were cost-effective HCC surveillance strategies compared with ultrasound alone, generating three times the Chinese 2021 gross domestic product per capita for patients with non-cirrhotic chronic HBV^43^. Additionally, a cost-utility analysis of a simulated cohort of 100,000 participants in the United Kingdom found that GAAD was cost-effective, compared with ultrasound alone and ultrasound combined with AFP measurements, in participants with compensated liver cirrhosis (CLC)^44^. In those with CLC and viral hepatitis etiology, GAAD remained dominant compared with ultrasound with AFP and more cost-effective than ultrasound alone^44^. Similarly, a Swiss study demonstrated that GAAD was the most cost-effective surveillance strategy compared with no surveillance, ultrasound alone, and ultrasound with AFP, in participants with CLC, non-cirrhotic fibrosis (stage 3), and non-cirrhotic chronic HBV^45^.

A large proportion of participants in this study were receiving anti-viral therapy for HBV (total HCC, 63.1%; benign controls, 61.0%), which is concurrent with the endemic nature of the virus within China^7,9^. It is important to note that elevated AFP serum levels in patients with HBV and HCV, in the absence of HCC, may also occur as a result of the transcriptional upregulation of AFP by the viral transcription co-regulator, HBx^33,46^. However, falsely elevated AFP in patients with active hepatitis would be minimized with antiviral therapy, through suppressing the mechanisms of viral replication, subsequently reducing inflammatory damage^33,47^. In addition, previous studies have demonstrated that a lower cut off for AFP displays high sensitivity for the detection of HCC among patients with HBV receiving anti-viral treatment^33,47^. Therefore, the addition of AFP to surveillance can further improve HCC detection in participants with HBV/HCV receiving antiviral therapies^33^. Considering this, further research incorporating additional populations is needed to clarify how each individual serum biomarker contributes to the performance of the GAAD and GALAD algorithms, accounting for disease etiology and any ongoing antiviral or other medical treatments.

### Limitations

Despite national vaccination programs, the majority of participants with HCC included in this Chinese study had viral-related etiology. This was expected as previous studies have shown that China has high rates of HBV and HCV, which account for 60% of HCC cases^7^. Although good clinical performance was observed across disease etiologies in our study, the limited numbers of participants with non-viral etiologies should be considered. However, the clinical validation of GAAD has been investigated in a larger cohort of patients with non-viral etiologies across the Asian-Pacific region and Europe with findings that support the results of the present study^36^. Another limitation of this study was the small sample sizes for disease groups in the specificity panel, which limited the number of disease groups with sufficient samples for analysis.

## Conclusions

Here, we show that GAAD and GALAD algorithms display similar clinical performance for the differentiation of benign CLD and HCC, irrespective of HCC disease stage, underlying etiology, or clinical study site. These findings suggest that the GAAD (Cobas) algorithm may be an efficient diagnostic tool in the surveillance of high-risk patients with CLD, including those with HBV, and could contribute to earlier HCC diagnoses, and therefore improved HCC outcomes.

## Electronic supplementary material

Below is the link to the electronic supplementary material.

Supplementary Material 1

## Data Availability

Requests concerning the data supporting the findings of this study can be directed to the corresponding author, Dr Ashish Sharma, or rotkreuz.datasharingrequests@roche.com for consideration.
