# Supplementary material for: Prospective appraisal of clinical diagnostic algorithms for hepatocellular carcinoma surveillance in Chinese patients with chronic hepatitis B infection
Source: Sci Rep. 2024 Nov 22;14:28996. doi: 10.1038/s41598-024-80257-w (PMC11584881; doi:10.1038/s41598-024-80257-w)
Supplement: Supplementary file 1 — Supplementary Material 1 [file 41598_2024_80257_MOESM1_ESM.docx]

**Prospective Appraisal of Clinical Diagnostic Algorithms for Hepatocellular Carcinoma Surveillance in Chinese Patients with Chronic Hepatitis B Infection**

**Supplementary Information**

**Supplementary Table S1.** Participant, disease, and tumor characteristics of the Chinese subpopulation by clinical study site.

| **Participant characteristics** | **Guangzhou (n=203)** | **Hong Kong (n=109)** |
| --- | --- | --- |
| Age, years, mean (SD) | 47.9 (11.0) | 58.3 (10.3) |
| Sex, n (%) |  |  |
| Male | 167 (82.3) | 78 (71.6) |
| Female | 36 (17.7) | 31 (28.4) |
| Race, n (%) |  |  |
| Asian | 202 (99.5) | 109 (100) |
| White/Caucasian | 0 | 0 |
| Black | 0 | 0 |
| Other | 1 (0.5) | 0 |
| Ongoing antiviral therapy, n (%) |  |  |
| Yes | 132 (65.0) | 62 (56.9) |
| No | 71 (35.0) | 47 (43.1) |
| Liver biochemistry (U/L), mean (SD) |  |  |
| AST | 45.8 (53.7) | 52.3 (61.3) |
| ALT | 40 (40.7) | 53.8 (66.3) |
| PT-INR, n (%)* |  |  |
| 1 | 119 (58.6) | 56 (51.4) |
| 2 | 0 | 0 |
| 3 | 1 (0.5) | 0 |
| Missing | 83 (40.9) | 53 (48.6) |
| Ascites, n (%) |  |  |
| Mild | 22 (10.8) | 3 (2.8) |
| Moderate to severe | 9 (4.4) | 2 (1.8) |
| None | 172 (84.7) | 104 (95.4) |
| Hepatic encephalopathy, n (%) |  |  |
| Grade I–II | 0 | 0 |
| None | 203 (100) | 109 (100) |
| Serum albumin (g/L), mean (SD) | 44.6 (40.1) | 37.8 (4.3) |
| Serum total bilirubin (μmol/L), mean (SD) | 20.6 (28.5) | 15.2 (10.8) |
| MELD score, mean (SD) | 7.8 (2.5) | 7.3 (1. 6) |
| ALBI score, mean (SD) | −3.0 (3.4) | −2.5 (0.5) |
| ALBI grade, n (%) |  |  |
| 1 | 127 (62.6) | 46 (42.2) |
| 2 | 71 (35.0) | 59 (54.1) |
| 3 | 5 (2.5) | 4 (3.7) |
| Disease etiology, n (%) |  |  |
| Cirrhosis | 88 | 27 |
| Cirrhotic HBV | 84 | 27 |
| Cirrhotic HCV | 3 | 1 |
| Cirrhotic MASH | 3 | 0 |
| Cirrhotic ALD | 0 | 1 |
| Cirrhotic other | 0 | 2 |
| Non-cirrhosis | 32 | 8 |
| Non-cirrhotic HBV | 32 | 8 |
| Non-cirrhotic HCV | 1 | 0 |
| Non-cirrhotic MASH | 2 | 0 |
| Non-cirrhotic ALD | 0 | 0 |
| Non-cirrhotic other | 0 | 0 |
| **Tumor characteristics** | **Guangzhou**  **(n=120)^†^** | **Hong Kong**  **(n=56)^†^** |
| PST, n (%) | 94 (78.3) | 46 (82.1) |
| 0 | 21 (17.5) | 10 (17.9) |
| 1 | 5 (4.2) | 0 |
| 2 |  |  |
| BCLC stage, n (%) | 20 (16.7) | 7 (12.5) |
| 0 | 47 (39.2) | 36 (64.3) |
| A | 19 (15.8) | 3 (5.4) |
| B | 32 (26.7) | 8 (14.3) |
| C | 2 (1.7) | 2 (3.6) |
| D |  |  |
| Child–Pugh class, n (%) | 91 (75.8) | 51 (91.1) |
| A | 27 (22.5) | 3 (5.4) |
| B | 2 (1.7) | 2 (3.6) |
| C |  |  |
| Nodule number, n (%) | 69 (57.5) | 42 (75.0) |
| 1 | 6 (5.0) | 9 (16.1) |
| 2 | 5 (4.2) | 1 (1.8) |
| ≥3 | 40 (33.3) | 4 (7.1) |
| Tumor characteristics, n (%) |  |  |
| Large multinodular | 21 (17.5) | 7 (12.5) |
| Portal vein invasion or EHS (N1, M1) | 32 (26.7) | 4 (7.1) |
| Single <2 cm | 23 (19.2) | 8 (14.3) |
| Single or ≤3 nodules ≤3 cm | 44 (36.7) | 37 (66.1) |
| Size of index lesion (cm), mean (SD) | 6.07 (4.4) | 4.0 (2.7) |

*Data were missing for all participants in the benign CLD cohort; †HCC cases only.

ALBI, albumin-bilirubin; ALD, alcoholic liver disease; ALT, alanine aminotransferase; AST, aspartate aminotransferase; BCLC, Barcelona Clinic Liver Cancer; CLD, chronic liver disease; EHS, extrahepatic spread; HBV, hepatitis B virus; HCV, hepatitis C virus; HCC, hepatocellular carcinoma; MELD, Model for End-Stage Liver Disease; MASH, metabolic dysfunction-associated steatohepatitis; PST, performance status test; PT-INR, prothrombin international normalized ratio; SD, standard deviation.

**Supplementary Table S2.** Clinical performance of GAAD (Cobas), GALAD (Cobas), GALAD (μTASWAKO), Elecsys AFP, Elecsys AFP-L3, and Elecsys PIVKA-II for the detection of early-, late-, and all-stage HCC at pre-defined cut-offs.

|  | **GAAD (Cobas)** | | | **GALAD (Cobas)** | | | **GALAD (****μTASWAKO)** | | | **Elecsys AFP** | | | **Elecsys AFP-L3** | | | **Elecsys PIVKA-II** | | |
| --- | --- | --- | --- | --- | --- | --- | --- | --- | --- | --- | --- | --- | --- | --- | --- | --- | --- | --- |
|  | **Early** | **Late** | **All** | **Early** | **Late** | **All** | **Early** | **Late** | **All** | **Early** | **Late** | **All** | **Early** | **Late** | **All** | **Early** | **Late** | **All** |
| N  (HCC/control) | 246 (110/136) | 202 (66/136) | 312 (176/136) | 246 (110/136) | 202 (66/136) | 312 (176/136) | 246 (110/136) | 202 (66/136) | 312 (176/136) | 246 (110/136) | 202 (66/136) | 312 (176/136) | 246 (110/136) | 202 (66/136) | 312 (176/136) | 246 (110/136) | 202 (66/136) | 312 (176/136) |
| TP | 74 | 62 | 136 | 73 | 62 | 135 | 63 | 54 | 117 | 50 | 49 | 99 | 49 | 51 | 100 | 57 | 59 | 116 |
| TN | 135 | 135 | 135 | 135 | 135 | 135 | 135 | 135 | 135 | 134 | 134 | 134 | 135 | 135 | 135 | 135 | 135 | 135 |
| FP | 1 | 1 | 1 | 1 | 1 | 1 | 0 | 0 | 0 | 2 | 2 | 2 | 1 | 1 | 1 | 1 | 1 | 1 |
| FN | 36 | 4 | 40 | 37 | 4 | 41 | 47 | 7 | 54 | 60 | 17 | 77 | 61 | 15 | 76 | 53 | 7 | 60 |
| Sensitivity  (95% CI) | 67.3(57.7–75.9) | 93.9 (85.2–98.3) | 77.3 (70.4–83.2) | 66.4 (56.7–75.1) | 93.9 (85.2–98.3) | 76.7 (69.8–82.7) | 57.3 (47.5–66.7) | 88.5 (77.8–95.3) | 68.4 (60.9–75.3) | 45.5 (35.9–55.2) | 74.2 (63.0–84.2) | 56.3 (48.6–63.7) | 44.5 (35.1–54.3) | 77.3 (65.3–86.7) | 56.8 (49.2–64.2) | 51.8 (42.1–61.4) | 89.4 (79.4–95.6) | 65.9 (58.4–72.9) |
| Specificity (95% CI) | 99.3 (96.0–100) | 99.3 (96.0–100) | 99.3 (96.0–100) | 99.3 (96.0–100) | 99.3 (96.0–100) | 99.3 (96.0–100) | 100 (97.3–100) | 100 (97.3–100) | 100 (97.3–100) | 98.5 (94.8–99.8) | 98.5 (94.8–99.8) | 98.5 (94.8–99.8) | 99.3 (96.0–100) | 99.3 (96.0–100) | 99.3 (96.0–100) | 99.3 (96.0–100) | 99.3 (96.0–100) | 99.3 (96.0–100) |
| PPV 1% | 48.0 | 56.3 | 51.5 | 47.7 | 56.3 | 51.3 | 100 | 100 | 100 | 23.8 | 33.8 | 27.9 | 38.0 | 51.5 | 43.8 | 41.6 | 55.1 | 47.5 |
| PPV 2% | 65.1 | 72.3 | 68.2 | 64.8 | 72.3 | 68.0 | 100 | 100 | 100 | 38.7 | 50.7 | 43.8 | 55.3 | 68.2 | 61.2 | 59.0 | 71.3 | 64.7 |
| PPV 3% | 73.9 | 79.8 | 76.5 | 73.6 | 79.8 | 76.3 | 100 | 100 | 100 | 48.9 | 61.0 | 54.2 | 65.2 | 76.5 | 70.5 | 68.5 | 79.0 | 73.5 |
| PPV 4% | 79.2 | 84.2 | 81.4 | 79.0 | 84.2 | 81.3 | 100 | 100 | 100 | 56.3 | 67.8 | 61.4 | 71.6 | 81.4 | 76.3 | 74.6 | 83.5 | 78.9 |
| PPV 5% | 82.8 | 87.1 | 84.7 | 82.6 | 87.1 | 84.6 | 100 | 100 | 100 | 61.9 | 72.7 | 66.8 | 76.1 | 84.7 | 80.3 | 78.8 | 86.5 | 82.5 |
| NPV 1% | 99.7 | 99.9 | 99.8 | 99.7 | 99.9 | 99.8 | 99.6 | 99.9 | 99.7 | 99.4 | 99.7 | 99.6 | 99.4 | 99.8 | 99.6 | 99.5 | 99.9 | 99.7 |
| NPV 2% | 99.3 | 99.9 | 99.5 | 99.3 | 99.9 | 99.5 | 99.1 | 99.8 | 99.4 | 98.9 | 99.5 | 99.1 | 98.9 | 99.5 | 99.1 | 99.0 | 99.8 | 99.3 |
| NPV 3% | 99.0 | 99.8 | 99.3 | 99.0 | 99.8 | 99.3 | 98.7 | 99.6 | 99.0 | 98.3 | 99.2 | 98.6 | 98.3 | 99.3 | 98.7 | 98.5 | 99.7 | 98.9 |
| NPV 4% | 98.6 | 99.7 | 99.1 | 98.6 | 99.7 | 99.0 | 98.3 | 99.5 | 98.7 | 97.7 | 98.9 | 98.2 | 97.7 | 99.1 | 98.2 | 98.0 | 99.6 | 98.6 |
| NPV 5% | 98.3 | 99.7 | 98.8 | 98.2 | 99.7 | 98.8 | 97.8 | 99.4 | 98.4 | 97.2 | 98.6 | 97.7 | 97.1 | 98.8 | 97.8 | 97.5 | 99.4 | 98.2 |

The cut-off for the clinical performance of GALAD (μTASWAKO) was defined as -0.69.

AFP, α-fetoprotein; AFP-L3, *Lens culinaris* agglutinin-reactive fraction of AFP; CI, confidence interval; FN, false negative; FP, false positive; GAAD, gender (biological sex), age, AFP, PIVKA-II; GALAD, gender (biological sex), age, AFP-L3, AFP, PIVKA-II; HCC, hepatocellular carcinoma; NPV, negative predictive value; PIVKA-II, protein induced by vitamin K absence or antagonist-II; PPV, positive predictive value; TN, true negative; TP, true positive.

**Supplementary Table S3**. Cut-offs values of Elecsys assays, AFP, AFP-L3 and PIVKA-II, and algorithmic scores for GAAD (Cobas) and GALAD (Cobas) at specified sensitivity.

| **AFP** | | | | | | | | | | | | | | | |
| --- | --- | --- | --- | --- | --- | --- | --- | --- | --- | --- | --- | --- | --- | --- | --- |
|  | **AFP** | **Sensitivity**  **(all-stage)** | **Sensitivity**  **(early-stage)** | **Sensitivity**  **(late-stage)** | **Specificity** | **PPV 1%** | **PPV 2%** | **PPV 3%** | **PPV 4%** | **PPV 5%** | **NPV 1%** | **NPV 2%** | **NPV 3%** | **NPV 4%** | **NPV 5%** |
| **Sensitivity 70%** | 7.77 | 70.5  (63.1–77.1) | 58.2 (48.4–67.5) | 90.9 (81.3–96.6) | 90.4 (84.2–94.8) | 6.93 | 13.1 | 18.6 | 23.5 | 28.0 | 99.7 | 99.3 | 99.0 | 98.7 | 98.3 |
| **Sensitivity 75%** | 5.85 | 75.0 (67.9–81.2) | 64.5 (54.9–73.4) | 92.4  (83.2–97.5) | 85.3 (78.2–90.8) | 4.90 | 9.43 | 13.6 | 17.5 | 21.2 | 99.7 | 99.4 | 99.1 | 98.8 | 98.5 |
| **Sensitivity 80%** | 5.00 | 80.1 (73.4–85.7) | 72.7 (63.4–80.8) | 92.4  (83.2–97.5) | 79.4 (71.6–85.9) | 3.78 | 7.36 | 10.7 | 14.0 | 17.0 | 99.7 | 99.5 | 99.2 | 99.0 | 98.7 |
| **Sensitivity 85%** | 3.79 | 85.2 (79.1–90.1) | 79.1 (70.3–86.3) | 95.5  (87.3–99.1) | 66.9 (58.3–74.7) | 2.54 | 4.99 | 7.38 | 9.69 | 11.9 | 99.8 | 99.6 | 99.3 | 99.1 | 98.9 |
| **Sensitivity 90%** | 3.34 | 90.3 (85.0–94.3) | 86.4 (78.5–92.2) | 97.0 (89.5–99.6) | 59.6 (50.8–67.9) | 2.21 | 4.36 | 6.46 | 8.52 | 10.5 | 99.8 | 99.7 | 99.5 | 99.3 | 99.2 |
| **Sensitivity 95%** | 2.12 | 95.5  (91.2–98.0) | 92.7 (86.2–96.8) | 100 (94.6–100) | 27.2 (19.9–35.5) | 1.31 | 2.61 | 3.90 | 5.18 | 6.46 | 99.8 | 99.7 | 99.5 | 99.3 | 99.1 |
| **PIVKA-II** | | | | | | | | | | | | | | | |
|  | **PIVKA-II** | **Sensitivity**  **(all-stage)** | **Sensitivity**  **(early-stage)** | **Sensitivity**  **(late-stage)** | **Specificity** | **PPV 1%** | **PPV 2%** | **PPV 3%** | **PPV 4%** | **PPV 5%** | **NPV 1%** | **NPV 2%** | **NPV 3%** | **NPV 4%** | **NPV 5%** |
| **Sensitivity 70%** | 26.2 | 70.5  (63.1–77.1) | 58.2 (48.4–67.5) | 90.9 (81.3–96.6) | 99.3 (96.0–100) | 49.2 | 66.2 | 74.8 | 80.0 | 83.5 | 99.7 | 99.4 | 99.1 | 98.8 | 98.5 |
| **Sensitivity 75%** | 18.9 | 75.0 (67.9–81.2) | 64.5 (54.9–73.4) | 92.4 (83.2–97.5) | 85.3 (78.2–90.8) | 4.90 | 9.43 | 13.6 | 17.5 | 21.2 | 99.7 | 99.4 | 99.1 | 98.8 | 98.5 |
| **Sensitivity 80%** | 17.1 | 79.5 (72.8–85.2) | 70.0 (60.5–78.4) | 95.5 (87.3–99.1) | 64.7 (56.1–72.7) | 2.23 | 4.4 | 6.52 | 8.58 | 10.6 | 99.7 | 99.4 | 99.0 | 98.7 | 98.4 |
| **Sensitivity 85%** | 16.3 | 85.2 (79.1–90.1) | 77.3  (68.3–84.7) | 98.5 (91.8–100) | 53.7 (44.9–62.3) | 1.82 | 3.62 | 5.38 | 7.12 | 8.83 | 99.7 | 99.4 | 99.2 | 98.9 | 98.6 |
| **Sensitivity 90%** | 15.1 | 89.8 (84.3–93.8) | 84.5 (76.4–90.7) | 98.5 (91.8–100) | 34.6 (26.6–43.2) | 1.37 | 2.72 | 4.07 | 5.41 | 6.73 | 99.7 | 99.4 | 99.1 | 98.8 | 98.5 |
| **Sensitivity 95%** | 13.5 | 94.3 (89.8–97.2) | 90.9 (83.9–95.6) | 100 (94.6–100) | 19.9 (13.5–27.6) | 1.17 | 2.35 | 3.51 | 4.67 | 5.83 | 99.7 | 99.4 | 99.1 | 98.8 | 98.5 |
| **AFP-L3** | | | | | | | | | | | | | | | |
|  | **AFP-L3** | **Sensitivity**  **(all-stage)** | **Sensitivity**  **(early-stage)** | **Sensitivity**  **(late-stage)** | **Specificity** | **PPV 1%** | **PPV 2%** | **PPV 3%** | **PPV 4%** | **PPV 5%** | **NPV 1%** | **NPV 2%** | **NPV 3%** | **NPV 4%** | **NPV 5%** |
| **Sensitivity 70%** | 1.2 | 100 (97.9–100) | 100 (96.7–100) | 100 (94.6–100) | 0  (0.00–2.68) | 1 | 2 | 3 | 4 | 5 | – | – | – | – | – |
| **Sensitivity 75%** | 1.2 | 100 (97.9–100) | 100 (96.7–100) | 100 (94.6–100) | 0  (0.00–2.68) | 1 | 2 | 3 | 4 | 5 | – | – | – | – | – |
| **Sensitivity 80%** | 1.2 | 100 (97.9–100) | 100 (96.7–100) | 100 (94.6–100) | 0  (0.00–2.68) | 1 | 2 | 3 | 4 | 5 | – | – | – | – | – |
| **Sensitivity 85%** | 1.2 | 100 (97.9–100) | 100 (96.7–100) | 100 (94.6–100) | 0  (0.00–2.68) | 1 | 2 | 3 | 4 | 5 | – | – | – | – | – |
| **Sensitivity 90%** | 1.2 | 100 (97.9–100) | 100 (96.7–100) | 100 (94.6–100) | 0  (0.00–2.68) | 1 | 2 | 3 | 4 | 5 | – | – | – | – | – |
| **Sensitivity 95%** | 1.2 | 100 (97.9–100) | 100 (96.7–100) | 100 (94.6–100) | 0  (0.00–2.68) | 1 | 2 | 3 | 4 | 5 | – | – | – | – | – |
| **GAAD** | | | | | | | | | | | | | | | |
|  | **GAAD** | **Sensitivity**  **(all-stage)** | **Sensitivity**  **(early-stage)** | **Sensitivity**  **(late-stage)** | **Specificity** | **PPV 1%** | **PPV 2%** | **PPV 3%** | **PPV 4%** | **PPV 5%** | **NPV 1%** | **NPV 2%** | **NPV 3%** | **NPV 4%** | **NPV 5%** |
| **Sensitivity 70%** | 4.02 | 70.5 (63.1–77.1) | 57.3 (47.5–66.7) | 92.4 (83.2–97.5) | 100 (97.3–100) | 100 | 100 | 100 | 100 | 100 | 99.7 | 99.4 | 99.1 | 98.8 | 98.5 |
| **Sensitivity 75%** | 3.09 | 75.0 (67.9–81.2) | 63.6 (53.9–72.6) | 93.9 (85.2–98.3) | 100 (97.3–100) | 100 | 100 | 100 | 100 | 100 | 99.7 | 99.5 | 99.2 | 99.0 | 98.7 |
| **Sensitivity 80%** | 1.86 | 80.1 (73.4–85.7) | 70.0 (60.5–78.4) | 97.0 (89.5–99.6) | 94.9 (89.7–97.9) | 13.6 | 24.1 | 32.5 | 39.3 | 45.0 | 99.8 | 99.6 | 99.4 | 99.1 | 98.9 |
| **Sensitivity 85%** | 1.16 | 85.2 (79.1–90.1) | 77.3 (68.3–84.7) | 98.5 (91.8–100) | 94.9 (89.7–97.9) | 14.3 | 25.3 | 33.9 | 40.8 | 46.6 | 99.8 | 99.7 | 99.5 | 99.4 | 99.2 |
| **Sensitivity 90%** | 0.878 | 90.3 (85.0–94.3) | 84.5 (76.4–90.7) | 100 (94.6–100) | 89.7 (83.3–94.3) | 8.14 | 15.2 | 21.3 | 26.8 | 31.6 | 99.9 | 99.8 | 99.7 | 99.6 | 99.4 |
| **Sensitivity 95%** | 0.469 | 95.5 (91.2–98.0) | 92.7 (86.2–96.8) | 100 (94.6–100) | 64.0 (55.3–72.0) | 2.61 | 5.13 | 7.57 | 9.94 | 12.2 | 99.9 | 99.9 | 99.8 | 99.7 | 99.6 |
| **GALAD** | | | | | | | | | | | | | | | |
|  | **GALAD** | **Sensitivity**  **(all-stage)** | **Sensitivity**  **(early-stage)** | **Sensitivity**  **(late-stage)** | **Specificity** | **PPV 1%** | **PPV 2%** | **PPV 3%** | **PPV 4%** | **PPV 5%** | **NPV 1%** | **NPV 2%** | **NPV 3%** | **NPV 4%** | **NPV 5%** |
| **Sensitivity 70%** | 4.24 | 70.5 (63.1–77.1) | 57.3 (47.5–66.7) | 92.4 (83.2–97.5) | 100 (97.3–100) | 100 | 100 | 100 | 100 | 100 | 99.7 | 99.4 | 99.1 | 98.8 | 98.5 |
| **Sensitivity 75%** | 3.11 | 75.0 (67.9–81.2) | 63.6 (53.9–72.6) | 93.9 (85.2–98.3) | 100 (97.3–100) | 100 | 100 | 100 | 100 | 100 | 99.7 | 99.5 | 99.2 | 99.0 | 98.7 |
| **Sensitivity 80%** | 1.91 | 79.5 (72.8–85.2) | 70 (60.5–78.4) | 95.5 (87.3–99.1) | 94.9 (89.7–97.9) | 13.5 | 24.0 | 32.3 | 39.2 | 44.9 | 99.8 | 99.6 | 99.3 | 99.1 | 98.9 |
| **Sensitivity 85%** | 1.22 | 85.2 (79.1–90.1) | 77.3 (68.3–84.7) | 98.5 (91.8–100) | 94.9 (89.7–97.9) | 14.3 | 25.3 | 33.9 | 40.8 | 46.6 | 99.8 | 99.7 | 99.5 | 99.4 | 99.2 |
| **Sensitivity 90%** | 0.946 | 90.3 (85.0–94.3) | 84.5 (76.4–90.7) | 100 (94.6–100) | 90.4  (84.2–94.8) | 8.71 | 16.2 | 22.6 | 28.3 | 33.2 | 99.9 | 99.8 | 99.7 | 99.6 | 99.4 |
| **Sensitivity 95%** | 0.497 | 95.5 (91.2–98.0) | 92.7 (86.2–96.8) | 100 (94.6–100) | 63.2 (54.5–71.3) | 2.56 | 5.03 | 7.43 | 9.76 | 12.0 | 99.9 | 99.9 | 99.8 | 99.7 | 99.6 |

AFP, α-fetoprotein; AFP-L3, *Lens culinaris* agglutinin-reactive fraction of AFP; GAAD, gender (biological sex), age, AFP, PIVKA-II; GALAD, gender (biological sex), age, AFP, AFP-L3, PIVKA-II; NPV, negative predictive value; PIVKA-II, protein induced by vitamin K absence or antagonist-II; PPV, positive predictive value.

**Supplementary Table S4.** Cut-off values of Elecsys assays, AFP, AFP-L3, and PIVKA-II, and algorithmic scores for GAAD (Cobas) and GALAD (Cobas) at specified specificity.

| **AFP** | | | | | | | | | | | | | | | |
| --- | --- | --- | --- | --- | --- | --- | --- | --- | --- | --- | --- | --- | --- | --- | --- |
|  | **AFP** | **Sensitivity**  **(all-stage)** | **Sensitivity**  **(early-stage)** | **Sensitivity**  **(late-stage)** | **Specificity** | **PPV 1%** | **PPV 2%** | **PPV 3%** | **PPV 4%** | **PPV 5%** | **NPV 1%** | **NPV 2%** | **NPV 3%** | **NPV 4%** | **NPV 5%** |
| **Specificity 70%** | 4.02 | 82.4 (75.9–87.7) | 76.4 (67.3–83.9) | 92.4  (83.2–97.5) | 69.9 (61.4–77.4) | 2.69 | 5.28 | 7.79 | 10.2 | 12.6 | 99.7 | 99.5 | 99.2 | 99.0 | 98.7 |
| **Specificity 75%** | 4.43 | 81.3 (74.7–86.7) | 74.5 (65.4–82.4) | 92.4  (83.2–97.5) | 75.0 (66.9–82.0) | 3.18 | 6.22 | 9.13 | 11.9 | 14.6 | 99.7 | 99.5 | 99.2 | 99.0 | 98.7 |
| **Specificity 80%** | 5.03 | 79.5 (72.8–85.2) | 71.8 (62.4–80.0) | 92.4  (83.2–97.5) | 79.4 (71.6–85.9) | 3.76 | 7.31 | 10.7 | 13.9 | 16.9 | 99.7 | 99.5 | 99.2 | 98.9 | 98.7 |
| **Specificity 85%** | 5.64 | 76.1 (69.1–82.2) | 66.4 (56.7–75.1) | 92.4  (83.2–97.5) | 84.6 (77.4–90.2) | 4.74 | 9.14 | 13.2 | 17.0 | 20.6 | 99.7 | 99.4 | 99.1 | 98.8 | 98.5 |
| **Specificity 90%** | 7.73 | 70.5 (63.1–77.1) | 58.2 (48.4–67.5) | 90.9 (81.3–96.6) | 89.7 (83.3–94.3) | 6.47 | 12.3 | 17.5 | 22.2 | 26.5 | 99.7 | 99.3 | 99.0 | 98.6 | 98.3 |
| **Specificity 95%** | 11.5 | 64.2 (56.6–71.3) | 54.5 (44.8–64.1) | 80.3 (68.7–89.1) | 94.9 (89.7–97.9) | 11.2 | 20.3 | 27.8 | 34.2 | 39.6 | 99.6 | 99.2 | 98.8 | 98.5 | 98.1 |
| **AFP-L3** | | | | | | | | | | | | | | | |
|  | **AFP-L3** | **Sensitivity**  **(all-stage)** | **Sensitivity**  **(early-stage)** | **Sensitivity**  **(late-stage)** | **Specificity** | **PPV 1%** | **PPV 2%** | **PPV 3%** | **PPV 4%** | **PPV 5%** | **NPV 1%** | **NPV 2%** | **NPV 3%** | **NPV 4%** | **NPV 5%** |
| **Specificity 70%** | 1.2 | 100 (97.9–100) | 100 (96.7–100) | 100 (94.6–100) | 0 (0–2.68) | 1 | 2 | 3 | 4 | 5 | – | – | – | – | – |
| **Specificity 75%** | 1.2 | 100 (97.9–100) | 100 (96.7–100) | 100 (94.6–100) | 0 (0–2.68) | 1 | 2 | 3 | 4 | 5 | – | – | – | – | – |
| **Specificity 80%** | 1.2 | 100 (97.9–100) | 100 (96.7–100) | 100 (94.6–100) | 0 (0–2.68) | 1 | 2 | 3 | 4 | 5 | – | – | – | – | – |
| **Specificity 85%** | 1.2 | 100 (97.9–100) | 100 (96.7–100) | 100 (94.6–100) | 0 (0–2.68) | 1 | 2 | 3 | 4 | 5 | – | – | – | – | – |
| **Specificity 90%** | 1.2 | 100 (97.9–100) | 100 (96.7–100) | 100 (94.6–100) | 0 (0–2.68) | 1 | 2 | 3 | 4 | 5 | – | – | – | – | – |
| **Specificity 95%** | 1.33 | 64.2 (56.6–71.3) | 51.8 (42.1–61.4) | 84.8 (73.9–92.5) | 94.9 (89.7–97.9) | 11.2 | 20.3 | 27.8 | 34.2 | 39.6 | 99.6 | 99.2 | 98.8 | 98.5 | 98.1 |
| **PIVKA-II** | | | | | | | | | | | | | | | |
|  | **PIVKA-II** | **Sensitivity**  **(all-stage)** | **Sensitivity**  **(early-stage)** | **Sensitivity**  **(late-stage)** | **Specificity** | **PPV 1%** | **PPV 2%** | **PPV 3%** | **PPV 4%** | **PPV 5%** | **NPV 1%** | **NPV 2%** | **NPV 3%** | **NPV 4%** | **NPV 5%** |
| **Specificity 70%** | 17.5 | 76.7 (69.8–82.7) | 67.3 (57.7–75.9) | 92.4 (83.2–97.5) | 69.1 (60.6–76.8) | 2.45 | 4.82 | 7.13 | 9.38 | 11.6 | 99.7 | 99.3 | 99.0 | 98.6 | 98.3 |
| **Specificity 75%** | 17.8 | 76.1 (69.1–82.2) | 66.4 (56.7–75.1) | 92.4 (83.2–97.5) | 74.3 (66.1–81.4) | 2.9 | 5.69 | 8.38 | 11.0 | 13.5 | 99.7 | 99.3 | 99.0 | 98.7 | 98.3 |
| **Specificity 80%** | 18.0 | 76.1 (69.1–82.2) | 66.4 (56.7–75.1) | 92.4 (83.2–97.5) | 80.1 (72.4–86.5) | 3.73 | 7.26 | 10.6 | 13.8 | 16.8 | 99.7 | 99.4 | 99.1 | 98.8 | 98.5 |
| **Specificity 85%** | 18.7 | 75.0 (67.9–81.2) | 64.5 (54.9–73.4) | 92.4 (83.2–97.5) | 84.6 (77.4–90.2) | 4.68 | 9.02 | 13.1 | 16.8 | 20.4 | 99.7 | 99.4 | 99.1 | 98.8 | 98.5 |
| **Specificity 90%** | 19.7 | 74.4 (67.3–80.7) | 63.6 (53.9–72.6) | 92.4 (83.2–97.5) | 89.7 (83.3–94.3) | 6.81 | 12.9 | 18.3 | 23.2 | 27.6 | 99.7 | 99.4 | 99.1 | 98.8 | 98.5 |
| **Specificity 95%** | 20.8 | 74.4 (67.3–80.7) | 63.6 (53.9–72.6) | 92.4 (83.2–97.5) | 94.9 (89.7–97.9) | 12.7 | 22.8 | 30.9 | 37.6 | 43.2 | 99.7 | 99.5 | 99.2 | 98.9 | 98.6 |
| **GAAD** | | | | | | | | | | | | | | | |
|  | **GAAD** | **Sensitivity**  **(all-stage)** | **Sensitivity**  **(early-stage)** | **Sensitivity**  **(late-stage)** | **Specificity** | **PPV 1%** | **PPV 2%** | **PPV 3%** | **PPV 4%** | **PPV 5%** | **NPV 1%** | **NPV 2%** | **NPV 3%** | **NPV 4%** | **NPV 5%** |
| **Specificity 70%** | 0.527 | 93.8 (89.1–96.8) | 90.0 (82.8–94.9) | 100 (94.6–100) | 69.9 (61.4–77.4) | 3.05 | 5.97 | 8.77 | 11.5 | 14.1 | 99.9 | 99.8 | 99.7 | 99.6 | 99.5 |
| **Specificity 75%** | 0.556 | 93.2 (88.4–96.4) | 89.1 (81.7–94.2) | 100 (94.6–100) | 75.0 (66.9–82.0) | 3.63 | 7.07 | 10.3 | 13.4 | 16.4 | 99.9 | 99.8 | 99.7 | 99.6 | 99.5 |
| **Specificity 80%** | 0.629 | 93.2 (88.4–96.4) | 89.1 (81.7–94.2) | 100 (94.6–100) | 80.1 (72.4–86.5) | 4.53 | 8.74 | 12.7 | 16.4 | 19.8 | 99.9 | 99.8 | 99.7 | 99.6 | 99.6 |
| **Specificity 85%** | 0.739 | 90.9 (85.7–94.7) | 85.5 (77.5–91.5) | 100 (94.6–100) | 84.6 (77.4–90.2) | 5.61 | 10.7 | 15.4 | 19.7 | 23.7 | 99.9 | 99.8 | 99.7 | 99.6 | 99.4 |
| **Specificity 90%** | 0.898 | 89.8 (84.3–93.8) | 83.6 (75.4–90.0) | 100 (94.6–100) | 89.7 (83.3–94.3) | 8.1 | 15.1 | 21.2 | 26.7 | 31.5 | 99.9 | 99.8 | 99.6 | 99.5 | 99.4 |
| **Specificity 95%** | 2.2 | 77.3 (70.4–83.2) | 67.3 (57.7–75.9) | 93.9 (85.2–98.3) | 95.6 (90.6–98.4) | 15.0 | 26.3 | 35.1 | 42.2 | 48.0 | 99.8 | 99.5 | 99.3 | 99.0 | 98.8 |
| **GALAD** | | | | | | | | | | | | | | | |
|  | **GALAD** | **Sensitivity**  **(all-stage)** | **Sensitivity**  **(early-stage)** | **Sensitivity**  **(late-stage)** | **Specificity** | **PPV 1%** | **PPV 2%** | **PPV 3%** | **PPV 4%** | **PPV 5%** | **NPV 1%** | **NPV 2%** | **NPV 3%** | **NPV 4%** | **NPV 5%** |
| **Specificity 70%** | 0.565 | 93.8 (89.1–96.8) | 90.0 (82.8–94.9) | 100 (94.6–100) | 70.6 (62.2–78.1) | 3.12 | 6.11 | 8.97 | 11.7 | 14.4 | 99.9 | 99.8 | 99.7 | 99.6 | 99.5 |
| **Specificity 75%** | 0.599 | 93.2 (88.4–96.4) | 89.1 (81.7–94.2) | 100 (94.6–100) | 75.0 (66.9–82.0) | 3.63 | 7.07 | 10.3 | 13.4 | 16.4 | 99.9 | 99.8 | 99.7 | 99.6 | 99.5 |
| **Specificity 80%** | 0.649 | 92.6 (87.7–96.0) | 88.2 (80.6–93.6) | 100 (94.6–100) | 80.1 (72.4–86.5) | 4.5 | 8.69 | 12.6 | 16.3 | 19.7 | 99.9 | 99.8 | 99.7 | 99.6 | 99.5 |
| **Specificity 85%** | 0.782 | 91.5 (86.3–95.2) | 86.4 (78.5–92.2) | 100 (94.6–100) | 85.3 (78.2–90.8) | 5.91 | 11.3 | 16.1 | 20.6 | 24.7 | 99.9 | 99.8 | 99.7 | 99.6 | 99.5 |
| **Specificity 90%** | 0.917 | 90.3 (85.0–94.3) | 84.5 (76.4–90.7) | 100 (94.6–100) | 89.7 (83.3–94.3) | 8.14 | 15.2 | 21.3 | 26.8 | 31.6 | 99.9 | 99.8 | 99.7 | 99.6 | 99.4 |
| **Specificity 95%** | 2.06 | 78.4 (71.6–84.2) | 68.2 (58.6–76.7) | 95.5 (87.3–99.1) | 94.9 (89.7–97.9) | 13.3 | 23.7 | 32.0 | 38.8 | 44.5 | 99.8 | 99.5 | 99.3 | 99.1 | 98.8 |

AFP, α-fetoprotein; AFP-L3, *Lens culinaris* agglutinin-reactive fraction of AFP; GAAD, gender (biological sex), age, AFP, PIVKA-II; GALAD, gender (biological sex), age, AFP, AFP-L3, PIVKA-II; NPV, negative predictive value; PIVKA-II, protein induced by vitamin K absence or antagonist-II; PPV, positive predictive value.
